# 380. Environmental Contamination with SARS-CoV-2 in Nursing Homes

**DOI:** 10.1093/ofid/ofab466.581

**Published:** 2021-12-04

**Authors:** Lona Mody, Kristen Gibson, Liza Bautista, Karen Neeb, Ana Montoya, Grace Jenq, John Mills, Lillian Min, Julia Mantey, Mohammed Kabeto, Andrzej Galecki, Marco Cassone, Emily T Martin

**Affiliations:** 1 University of Michigan, Ann Arbor, Michigan; 2 University of Michigan School of Medicine, Ann Arbor, MI

## Abstract

**Background:**

The COVID-19 pandemic has disproportionately affected nursing home (NH) patients, accounting for 5% of all cases and 32% of all COVID-19 deaths nationwide. Little is known about the frequency and persistence of SARS-CoV-2 environmental contamination in NHs. We characterize SARS-CoV-2 contamination in the rooms of COVID-19 patients and common areas in and around COVID-19 units.

**Methods:**

A prospective cohort study was conducted at four NHs in Michigan between October 2020 and January 2021. Clinical research personnel obtained swab specimens from high-touch room surfaces of COVID-19 infected patients, up to three times per patient. Weekly swab specimens from six high-touch surfaces in common areas were also obtained. Demographic and clinical data were collected from patient clinical records. Our primary outcome of interest was the probability of SARS-CoV-2 detection from specific environmental surfaces in COVID-19 patient rooms.

**Results:**

One hundred four patients with COVID-19 were enrolled and followed for 241 visits. Patient characteristics included: 61.5% over the age of 80; 67.3% female; 89.4% non-Hispanic white; 50.1% short-stay. The study population had significant disabilities in activities of daily living (ADL; 81.7% dependent in four or more ADLs) and comorbidities including dementia (55.8%), diabetes (40.4%) and heart failure (32.7) (Table 1). Over the 3-month study period, 2087 swab specimens were collected (1896 COVID-19 patient room surfaces, 191 common area swabs). Figure 1 shows contamination rates at sites proximate and distant to the patient bed. SARS-CoV-2 positivity was 28.4% (538/1896 swabs) on patient room surfaces and 3.7% (7/191 swabs) on common area surfaces. Over the course of follow-up, 89.4% (93/104) of patients had SARS-CoV-2 contamination in their room at least once (Figure 2). Environmental contamination detected on enrollment correlated with contamination of the same site during follow-up. Functional independence increased the odds of proximate contamination.

Table 1. Clinical and Demographic Characteristics of the Study Population Including Short- and Long-stay Patients

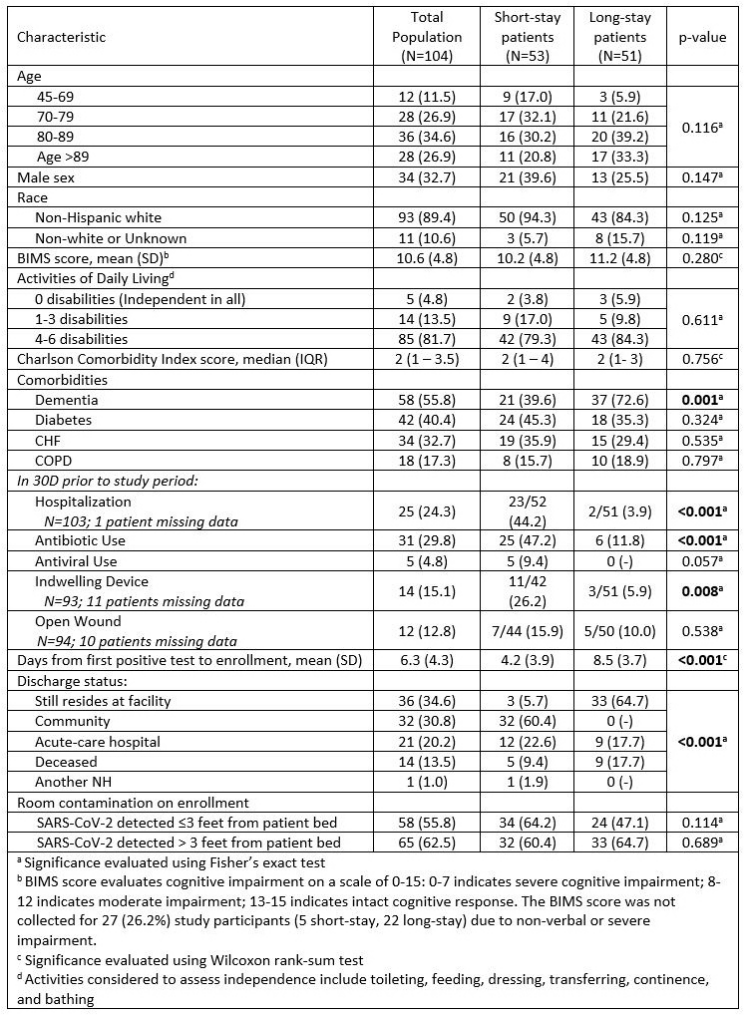

Figure 1. Contamination of Environmental Surfaces Relative to Distance from Patient Bed

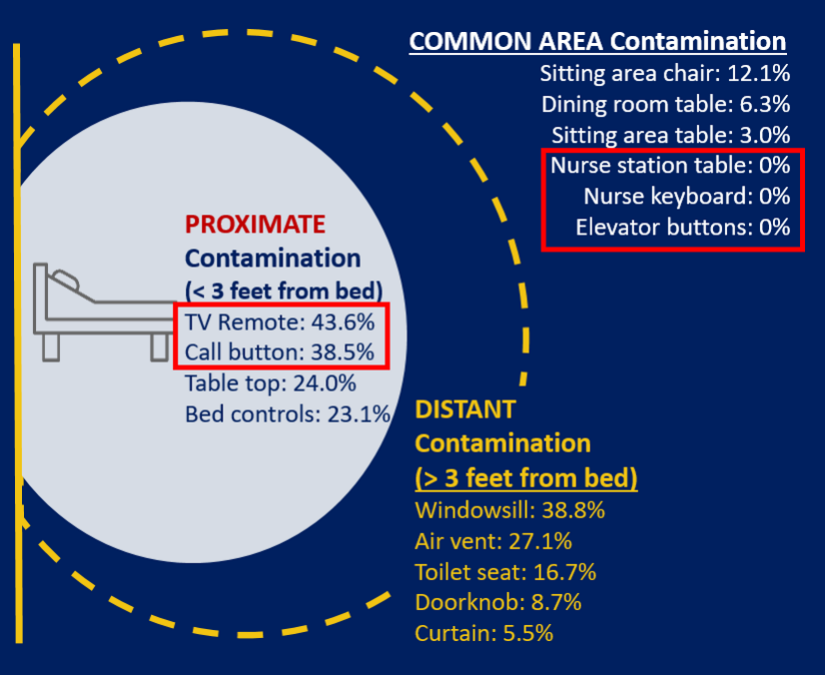

Figure 2. SARS-CoV-2 on Swab Specimens Collected – Patient-level, Visit-level, and Swab-level

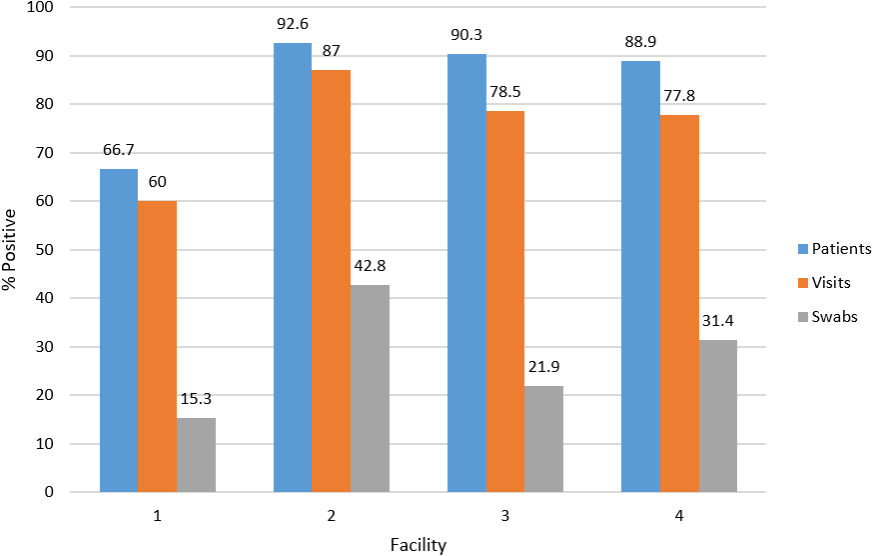

**Conclusion:**

We conclude that environmental contamination of surfaces in the rooms of COVID-19 patients is nearly universal and persistent. Patients with greater independence are more likely than fully dependent patients to contaminate their immediate environment.

**Disclosures:**

**All Authors**: No reported disclosures

